# The origin and insecticide resistance of *Aedes albopictus* mosquitoes established in southern Mozambique

**DOI:** 10.1186/s13071-024-06375-6

**Published:** 2024-07-08

**Authors:** Sarina Yamashita, Kawane Uruma, Chao Yang, Yukiko Higa, Noboru Minakawa, Nelson Cuamba, Kyoko Futami

**Affiliations:** 1 School of Medicine, Nagasaki University, 1-12-4 Sakamoto, Nagasaki, 852-8523 Japan; 2 Department of Medical Entomology, National Institute of Infectious Diseases, Toyama 1-23-1, Shinkuku-Ku, Tokyo, 162-8640 Japan; 3 Department of Vector Ecology & Environment, Institute of Tropical Medicine, Nagasaki University, 1-12-4 Sakamoto, Nagasaki, 852-8523 Japan; 4 Instituto Nacional de Saúde, Ministério da Saúde, C.P. 264, Maputo, Mozambique

**Keywords:** *Aedes albopictus*, Mozambique, Invasion, COI, Microsatellites, *kdr* mutation

## Abstract

**Background:**

The *Aedes albopictus* mosquito is of medical concern due to its ability to transmit viral diseases, such as dengue and chikungunya. *Aedes albopictus* originated in Asia and is now present on all continents, with the exception of Antarctica. In Mozambique, *Ae. albopictus* was first reported in 2015 within the capital city of Maputo, and by 2019, it had become established in the surrounding area. It was suspected that the mosquito population originated in Madagascar or islands of the Western Indian Ocean (IWIO). The aim of this study was to determine its origin. Given the risk of spreading insecticide resistance, we also examined relevant mutations in the voltage-sensitive sodium channel (VSSC).

**Methods:**

Eggs of *Ae. albopictus* were collected in Matola-Rio, a municipality adjacent to Maputo, and reared to adults in the laboratory. Cytochrome* c* oxidase subunit I (COI) sequences and microsatellite loci were analyzed to estimate origins. The presence of knockdown resistance (*kdr*) mutations within domain II and III of the VSSC were examined using Sanger sequencing.

**Results:**

The COI network analysis denied the hypothesis that the *Ae. albopictus* population originated in Madagascar or IWIO; rather both the COI network and microsatellites analyses showed that the population was genetically similar to those in continental Southeast Asia and Hangzhou, China. Sanger sequencing determined the presence of the F1534C knockdown mutation, which is widely distributed among Asian populations, with a high allele frequency (46%).

**Conclusions:**

These results do not support the hypothesis that the Mozambique *Ae. albopictus* population originated in Madagascar or IWIO. Instead, they suggest that the origin is continental Southeast Asia or a coastal town in China.

**Graphical Abstract:**

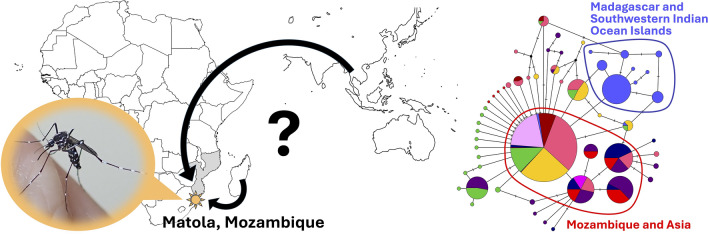

**Supplementary Information:**

The online version contains supplementary material available at 10.1186/s13071-024-06375-6.

## Background

Globalization is leading to the worldwide expansion of various cosmopolitan insects, including infectious disease vectors such as mosquitoes and ticks. *Aedes albopictus* is a cosmopolitan mosquito that originated in Asia and which transmits globally prevalent arboviruses such as dengue (DENV), chikungunya (CHIKV) and Zika (ZIKV).

In recent years, the introduction and expansion of *Ae. albopictus *populations in African countries has become a tremendous public health concern. This mosquito was first reported in the African continent in 1989, in Cape Town, South Africa [1], with immature stages of *Ae. albopictus* found in tires imported from Japan in 1989 and 1990 [1]. However, effective control prevented mosquito establishment in the country. In 1991, eggs of *Ae. albopictus *were collected by ovitraps in Delta State, Nigeria, which is the first record of a breeding population in the African continent [2]. *Aedes albopictus* was also reported from South Africa in 1992, but it failed to establish a population [3]. Since then, *Ae. albopictus* has established local populations in six West African countries [4–9] and was found to be a principal vector in CHIKV, DENV, and ZIKV outbreaks in Gabon, Cameroon and the Democratic Republic of the Congo (DRC) [10].

Before its introduction to the African continent, *Ae. albopictus* had established in the islands of the Western Indian Ocean (IWIO), being first reported in Mauritius in 1900, followed by Madagascar in 1904, Seychelles in 1912, La Réunion in 1913, Rodrigues in 1923, Mayotte in 2001 and Glorieuse in 2008 [11]. In IWIO, Chikungunya outbreaks caused by CHIKV occurred from 2005 to 2006, and then co-circulation of DENV was also reported [12–14]. *Aedes albopictus* has been implicated as the primary vector of these arboviruses in these outbreaks [13, 15–18], as this mosquito has high vector competence for the E1-226V strain of CHIKV, which has an amino acid mutation within the envelope protein [17–23]. However, the vector competence of *Ae. albopictus* varies among geographical populations and virus species [14, 19, 20, 24–26].

The introduction of a vector population may bring unwelcome traits, and thus identifying vector origins is essential to estimating the risk. Knockdown resistance (*kdr*) mutations have been reported within the *Ae. albopictus* population in West Africa, although their frequency is still low among the established populations [27–33]. *kdr* mutations of this species have been observed at three loci within the voltage-sensitive sodium channel (VSSC) domains II (V1016G in DII) and III (I1532T and F1534C/S/L in DIII). The mutation at codon 1534, which is associated with resistance to pyrethroid insecticides, has often been observed worldwide [29, 30, 32, 34–39], and the introduction of these resistant alleles is of concern for vector control in African countries [40]. If the introduced *Ae. albopictus* population originated in the temperate region, the overwintering ability of the temperate population may expand its distribution toward cooler areas [41].

Mitochondrial DNA (mtDNA) studies suggest that the West and Central African *Ae. albopictus* populations have multiple tropical and temperate origins [42–44]. A study using cytochrome* c* oxidase subunit I (COI) revealed that the Madagascar and the La Réunion populations are close to those of East Asia and North America [45]. In contrast, microsatellite studies have shown that the La Réunion population is similar to those of Southeast Asia and North America [45, 46]. The discrepancy between the two molecular studies of the La Réunion population is still the subject of debate [45].

In 2015, adults of *Ae. albopictus* were collected in Maputo, the capital of Mozambique [47]. A broader geographical survey in 2016 also found *Ae. albopictus* in Tete, located in the central region of Mozambique [48]. In 2018, large numbers of this mosquito species were collected using ovitraps at several sites within Matola-Rio, a municipality adjacent to Maputo. These findings confirmed that this species was established in the region, and represented its first recorded first establishment in the southern and eastern African regions.

The aim of the present study was to determine the origin of *Ae. albopictus* and to identify its insecticide resistance status to better understand the potential health risks associated with this established population. The study hypothesis was that *Ae. albopictus* originated from established populations in the nearby islands, Madagascar or IWIO. We also examined the occurrence of the *kdr* mutations in VSSC DII and DIII.

## Methods

### Mosquito collection

**Fig. 1:**
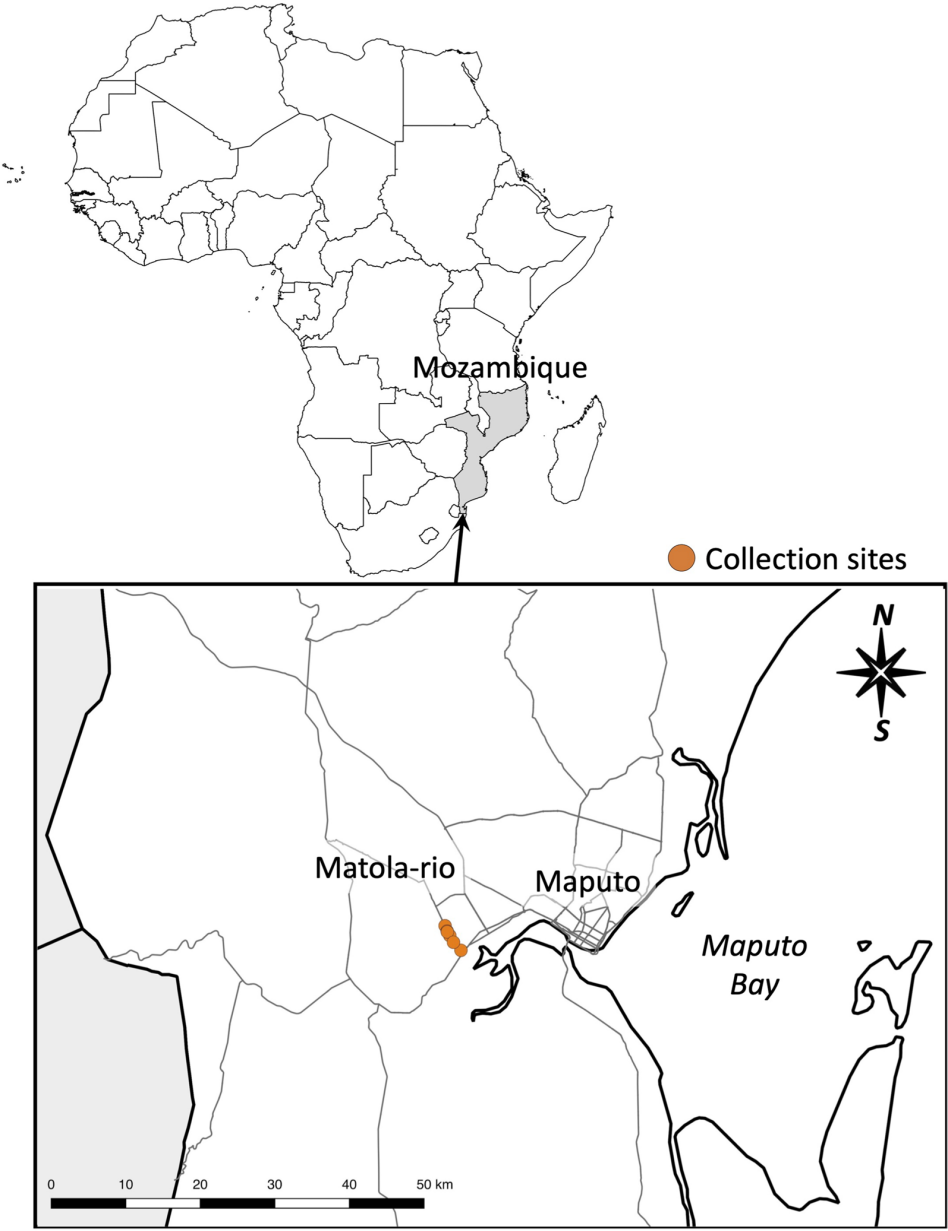
Ovitrap sites for *Aedes albopictus* in Matola-Rio, Boane district in Maputo province in Mozambique

Mosquitoes were sampled in Matola-Rio in Boane district, Maputo province in southern Mozambique (Fig. 1). The area is classified as a savanna climate with the Köppen climate classification [49]. Four to ten ovitraps were placed at each of six sites (4 restaurants and 1 garage) along 4 km of the Mozal Road in February and March 2019. The collected eggs were dried and transferred to the Institute of Tropical Medicine, Nagasaki University, Nagasaki, Japan. All collected eggs were mixed and reared together to pupae in an environmental chamber under conditions of 25 °C, 70% relative humidity and a photoregimen of 16/8 h (light/dark). The pupae were divided into separate cages by sex. Emerging adults were morphologically examined, and only *Ae. albopictus* adults were used in subsequent genetic analyses.

### DNA extraction

DNA was extracted from a single leg of randomly selected mosquitoes (25 females, 25 males) using a REDExtract-N-Amp Tissue PCR Kit (Merck KGaA, Darmstadt, Germany) following the manufacturer’s protocol.

### Determination of COI sequences and haplotype network

Partial COI sequences were amplified using two sets of primers (albo1454F and albo2160R; albo2027F and albo2886R) [50]. Each PCR analysis was performed in a 10 µl reaction volume containing 1.0 µl of template DNA, 3.6 µl distilled water, 5.0 µl REDExtract-N-AmpTMPCR Reaction Mix and 0.2 µl of each of the forward and reverse primers (mentioned in previous sentence). PCR thermocycling included an initial denaturing step at 94 °C for 3 min, followed by 35 cycles of 94 °C for 30 s, 55 °C for 30 s and 72 °C for 1 min, with a final additional extension at 72 °C for 6 min. The amplicons were used for cycle sequencing reaction with the BigDye Terminator v3.1 Kit (Thermo Fisher Scientific, Waltham, MA, USA) and sequenced on an ABI3730 DNA analyzer (Thermo Fisher Scientific). The sequences were aligned with MEGA X software [51] and haplotypes were confirmed. The number of haplotypes, haplotype diversity, nucleotide diversity, Fu’s Fs and Tajima’s D were calculated using the DnaSP version 6.12.03 software package [52]. To estimate the genetic relationship of the global population, we obtained COI haplotypes in IWIO, Madagascar, Oceania, Asia, Americas, the Middle East, the African continent and Europe (Additional file 1: Table S1) [11, 50, 53–66] and constructed TCS haplotype network [67] using PopART-1.7 [68] with longer (1302 bp) and shorter (452 bp) lengths.

### Genotyping of microsatellites loci and Bayesian clustering

Thirteen microsatellites loci that had been previously determined [69] were genotyped for the 50 DNA samples following Yang et al. [58]. For all loci, the allelic richness (A), expected heterozygosity (He), observed heterozygosity (Ho) and inbreeding coefficient (Gis) were calculated using GenoDive [70]. The obtained genotype data were analyzed together with previously reported data from Japan, the Philippines and Thailand [58]. The genetic differences between each pair of populations (Fst) were calculated using GenoDive. Bayesian clustering was done with STRUCTURE, and the analyzed results were visualized by CLAMPAC [71]. Based on a pre-run, the number of clusters (K) was set from 1 to 19. Following Yang et al. [58], each run consisted of 200,000 burn-in replications followed by 1,000,000 samplings. Collection locations were used as prior information, and an allele frequency correlated model was applied for 10 independent runs as replication. The best K was determined based on Evanno’s criteria for delta K [72]. The posterior probability of individual assignment to each cluster was rearranged by selecting the best K using DISTRUCT on the CLUMPACK server. Discriminant analysis of principal components (DAPC) was applied for the data using the package ‘adegenet’ of R to show similarity among the populations [73].

### Detection of point mutations in the VSSC gene

**Fig. 2:**
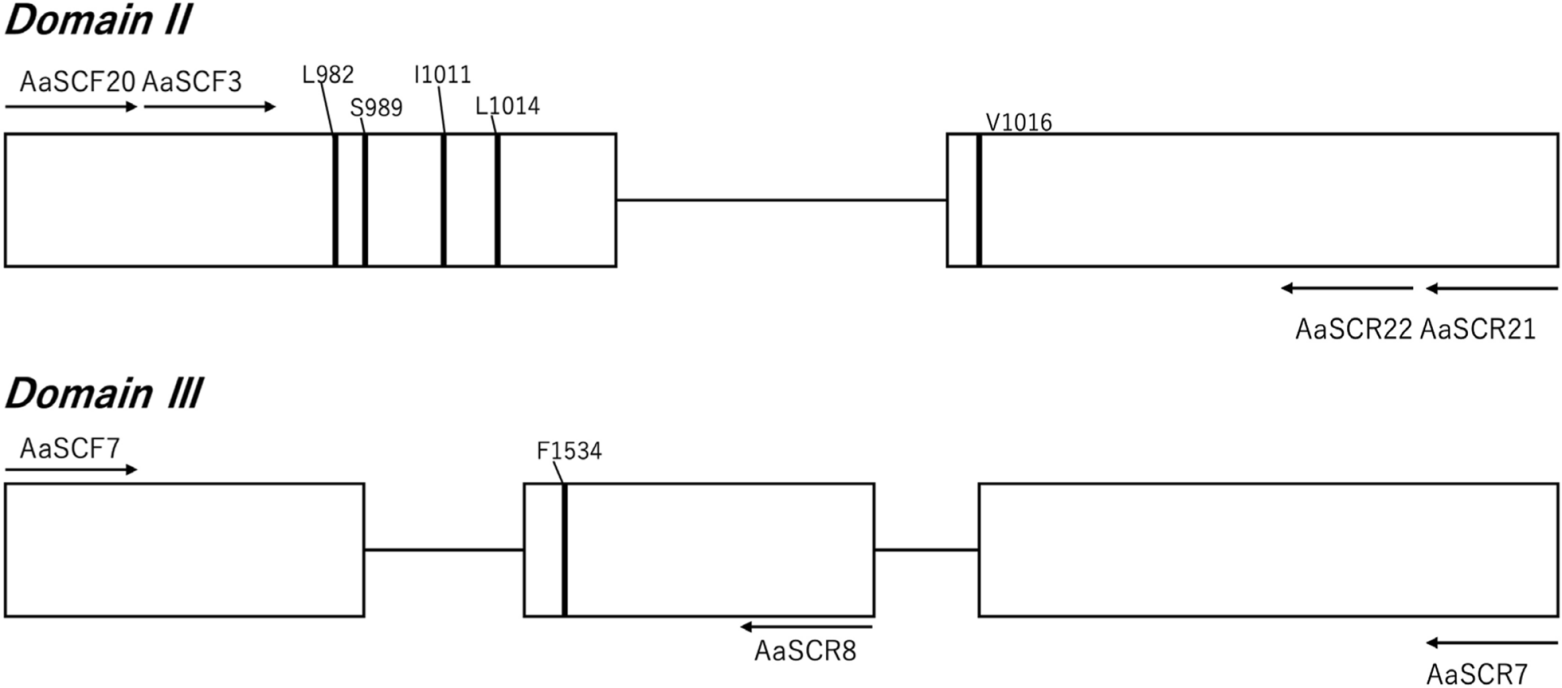
Diagram of the location of possible *kdr* mutations and primer position in domain II and domain III of the voltage-sensitive sodium channel gene. Rectangles indicate exons and solid lines indicate introns. Vertical lines drawn in rectangles indicate* kdr* mutations found in past studies. Arrows show the direction of the primer with the name written above the arrow.* kdr* Knockdown resistance

Six amino acid loci were targeted to identify *kdr* candidate mutations (DII: L982, S989, I1011, L1013, V1016; DIII: F1534). The amplification PCR for DII was performed in a total volume of 10 µl using 6.34 µl of double-distilled water, 0.06 µl of 10× Ex Taq HS (Takara bio, Shiga, Japan), 1.0 µl of 10× Ex Taq Buffer, 0.8 µl of 2.5 mM dNTP Mixture, 0.4 µl of each primer for DII (AaSCF20, 10 µM [5′-GTGGATCGCTTCCC-3′] and AaSCR21, 10 µM [5′-GCAATCTGGGCTTGTTAACTTG-3′] (Fig. 2) and 1.0 µl of DNA template. PCR amplification for DIII was performed using the primer set AaSCF7 (5′-GAGAACTCGCCGATGAACTT-3′) and AaSCR7 (5′-GACGACGAAATCGAACAGGT-3′) [29] (Fig. 2). The PCR amplification regimen consisted of 3 min at 94 ºC, followed by 35 cycles of 94 ºC for 15 s, 55 ºC for 30 s and 72 ºC for 30 s, with a final extension at 72 ºC for 10 min. The PCR products were cleaned using Exo-SAP-IT (Thermo Fisher Scientific) and then sequenced using the BigDye Terminator v3.1 Kit. The reaction mix contained 1.0 µl of the cleaned product, 0.34 µl of Big Dye terminator, 2.0 µl of 5× Sequencing Buffer, 5.66 µl of distilled water and 1.0 µl of one of the three primers (at 10 µM) (Fig. 2): AaSCF3 (5′-GTGGAACTTCACCGACTTCA-3′) and AaSCR22 (5′-TTCACGAACTTGAGCGCGTTG-3′) for DII and AaSCR8 (5′-TAGCTTTCAGCGGCTTCTTC-3′) for DIII. The reaction cycle followed the manufacturer’s protocol. The PCR products were purified by ethanol precipitation, dissolved in Hi-Di Formamide and sequenced on an ABI 3500 sequencer (Thermo Fisher Scientific). The determined sequences were aligned with MEGA X and searched for *kdr* mutations.

## Results

### COI sequences and haplotype network

The amplified mitochondrial COI gene spanned an aligned length of 1302 bp, within which seven variable sites were observed, five of which were parsimony-informative sites (GenBank accession numbers: LC726376–LC726425). Eight haplotypes were determined, all of which have been registered in GenBank. The haplotype diversity of the Matola-Rio population was 0.77, and the nucleotide diversity was < 0.01. The values of Tajima’s D and Fu’s Fs were not statistically significant (D = 0.11,* P* = 0.61, two-tailed test based on the beta distribution; Fs = − 1.14,* P* = 0.30, coalescent simulation), which indicated no selection pressure on the population.

**Fig. 3﻿:**
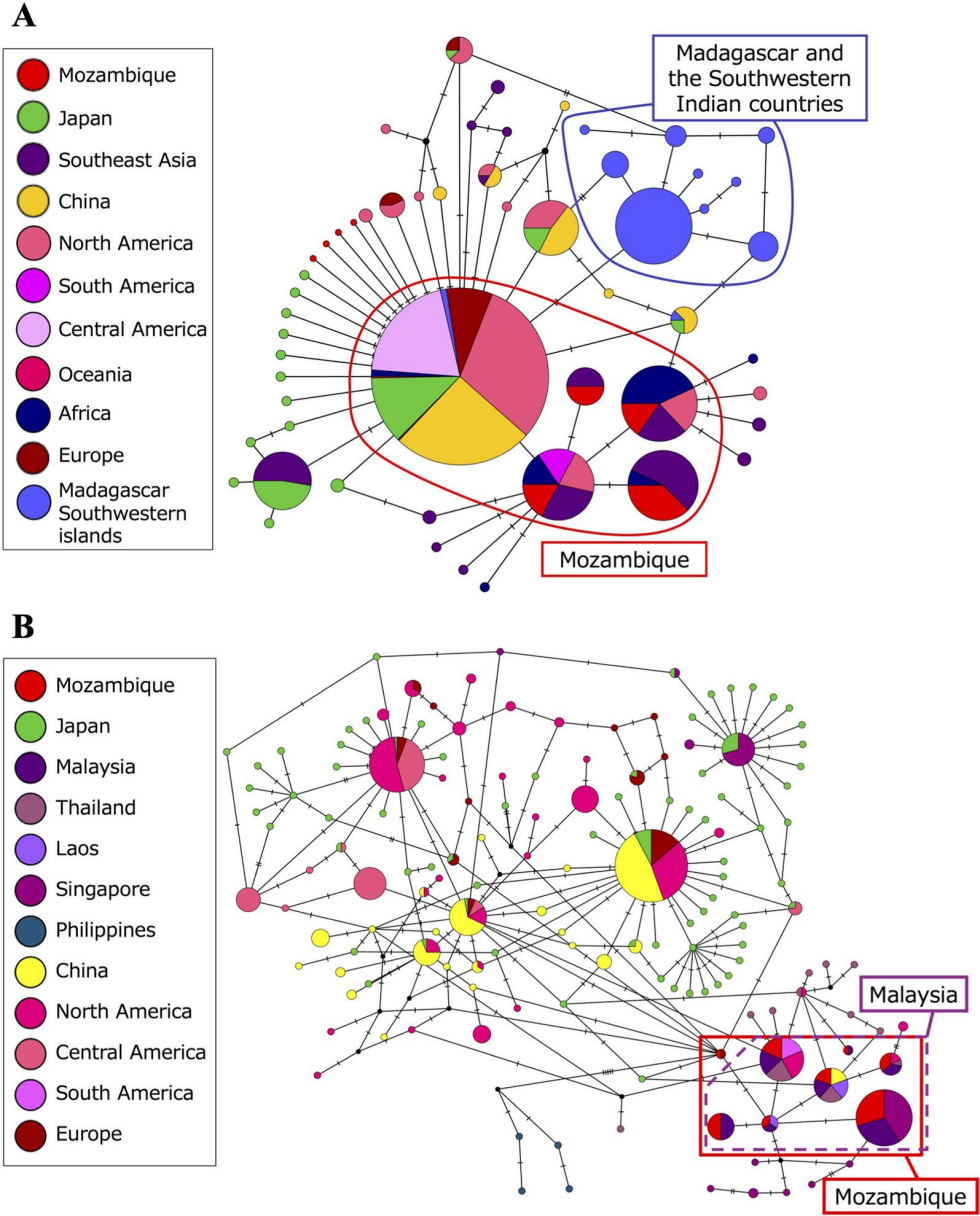
Haplotype networks drawn with COI sequences of *Aedes albopictus* collected in Matola-Rio, Mozambique. **A** Network constructed with 425-bp sequences, including sequences from Western Indian Ocean Islands and Madagascar populations. **B** Network constructed with 1302-bp sequences.* COI* Cytochrome c oxidase subunit I

To compare to the Madagascar population, the sequences were shortened, and the eight haplotypes were consolidated into five. Four hyprotypes found in the Matola-Rio population did not match with those found in the Madagascar and IWIO populations (Fig. 3a). One haplotype was shared with those populations, but it was a cosmopolitan haplotype (Fig. 3a). Analysis of the longer sequences revealed that seven of the eight haplotypes matched with those discovered in the Malaysia population (Fig. 3b). Some of these were shared with populations identified in Singapore and Hangzhou, China (Fig. 3b).

### Microsatellites loci and Bayesian clustering

**Fig. 5:**
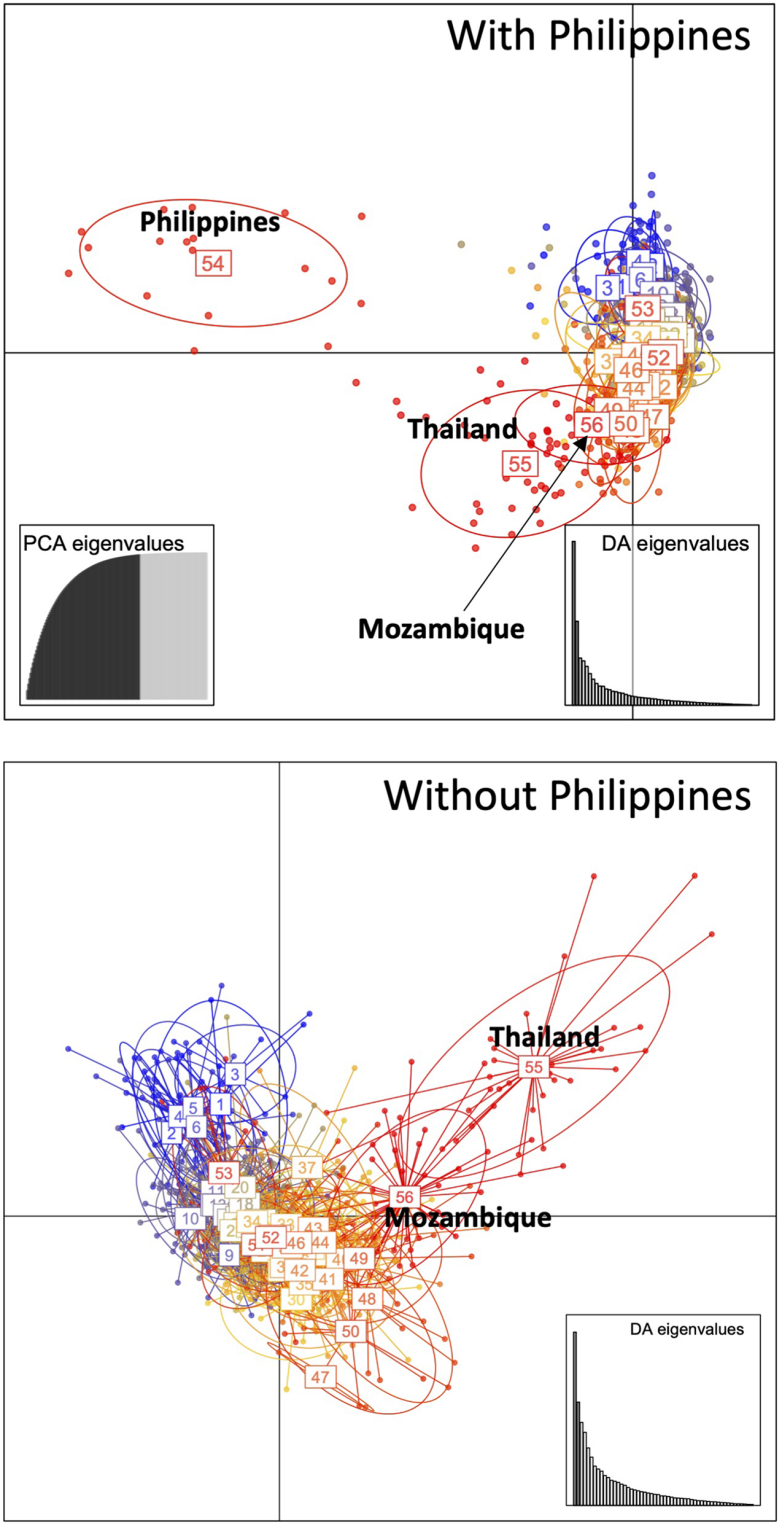
Results of DAPC of microsatellites genotypes of *Aedes albopictus* collected in Matola-Rio, Mozambique. DAPC, Discriminant analysis of principal component

Microsatellite analyses of 13 loci revealed that the values of A, Ho, He and Gis were 5.62, 0.50, 0.65 and 0.24, respectively (Additional file 2: Table S2). Compared to the other populations [58], the Matola-Rio population showed relatively low Gis, suggesting that the inbreeding was not strong. Pairwise Fst between the Matola-Rio population and the other populations was between 0.10 and 0.62, and the Tsushima and Nagasaki populations were relatively similar (Additional file 3: Table S3; Additional file 4: Table S4). The best delta K was 2, but delta Ks of 3, 7 and 10 also showed marked peaks (Fig. 4). The results of clustering with those Ks showed that the Matola-Rio population was: (i) genetically different from the representative populations of temperate regions collected in Fukuoka, Japan (K = 2); (ii) more similar to the Ryukyu and Thailand populations than the Nagasaki, Goto and Tsushima populations; and (iii) closer to the Thailand population than the other populations (Fig. 4). Since the Philippines population was distinct from the other populations in DAPC (Additional file 5: Data S5; Additional file 6: R command S6), the population was removed, and the data reanalyzed (Fig. 5a). The biplot graph showed that the Matola-Rio population was placed between the Thailand and Japanese populations (Fig. 5b).

**﻿Fig. 4:**
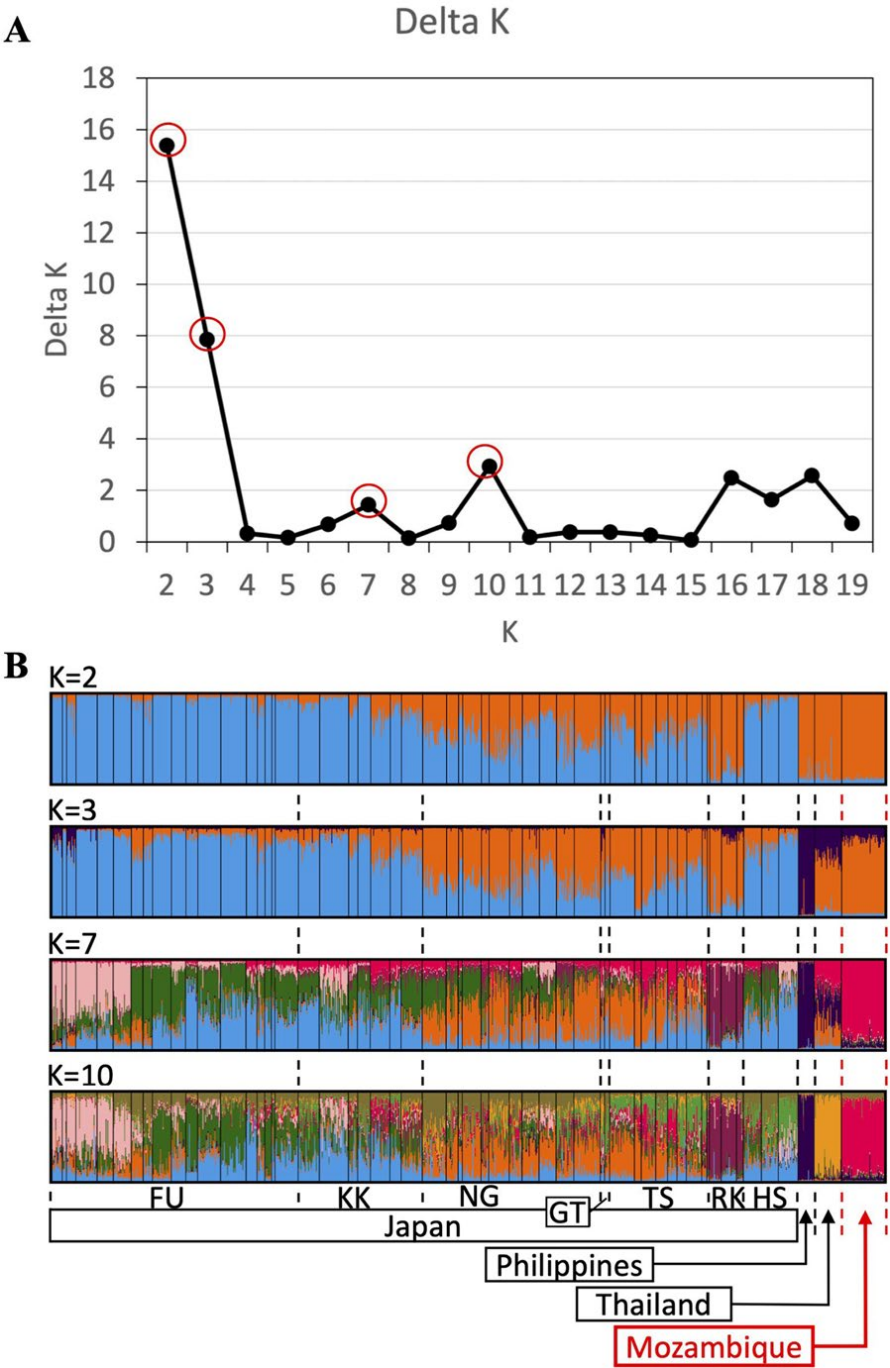
The delta Ks and posterior probability of belonging to an inferred cluster by Bayesian clustering with microsatellite genotypes of *Aedes albopictus* collected in Matola-Rio, Mozambique. **A** Delta Ks. **B** Posterior probability of individuals. The abbreviations FU, KK, NG, GT, TS, RK and HS are population names following Yang et al. [58]

### Detection of point mutations in the VSSC gene

A *kdr* mutation from phenylalanine to cysteine at 1534 in DIII was found in 29 individuals (Table 1). Of 44 samples successfully sequenced, 11 (25%) were found to be homozygous for F1534C. The gene frequency of 1534C was 46% (40/88). Mutations were not found in DII for all 50 individuals (Table 1).

**Table 1:** The number and frequencies of genotypes within the voltage-gated sodium channel gene in *Aedes albopictus* collected in Mozambique

Number and frequencies of genotypes	Loci of amino acids of VSSC							
	L982	S989	I1011	L1014	V1016	F1534		
Genotype^a^	LL	SS	II	LL	VV	FF	FC	CC
No. of samples	50 (50)	50 (50)	50 (50)	50 (50)	50 (50)	15 (44)	18 (44)	11 (44)
Frequency	1.0	1.0	1.0	1.0	1.0	0.34	0.41	0.25

The numbers in parentheses indicate the total number of successfully sequenced samples

^a^The capital letters are abbreviations of amino acids where L = leucine, S = serine, I = isoleucine, V = valine, F = phenylalanine, C = cysteine)

## Discussion

The results from the present study do not support the hypothesis that the Matola-Rio *Ae. albopictus* population was introduced from the long-established population in Madagascar or IWIO [11]. In contrast, the COI analyses showed that the Matola-Rio population shared haplotypes with tropical Asian populations—specifically, Singapore, Malaysia, and China.

The results of the COI network analyses suggested that the Matola-Rio population is closer to the Hangzhou population in the temperate area (Fig. 3). On the other hand, the microsatellite analysis revealed that the Matola-Rio population is not related to the populations in Japan that are also located the temperate area. The microsatellite analyses also showed that the Matola-Rio population is closer to the Thailand population than is the Philippines population. The results from the microsatellite analyses are comparable to ones from the COI analysis; specifically, the Malaysian population shares more haplotypes with the Thailand population than the Philippines population and the Japanese population. These results suggest that the Matola-Rio population is closely related to continental Southeast Asia and a coastal city of China, Hangzhou.

These results from the genetic analyses imply that the Matola-Rio population was introduced by ships from continental Southeast Asia and coastal China. According to the trade statistics of Mozambique in 2019, provided by the Observatory of Economic Complexity (OEC) [74], the largest importing partner (13.3% of total import value) was China, followed by India (12.8%), the DRC (3.8%), Singapore (3.8%), Malaysia (1.6%) and Japan (1.6%) (https://oec.world/en/profile/country/moz?yearlyTradeFlowSelector=flow1&yearSelector1=2019 (accessed 21 Sept 2023). Both Maputo city and Matola-Rio host the country’s largest harbor. These two pieces of information support the introduction of the Matola-Rio *Ae. albopictus* population from Hangzhou, Singapore and Malaysia. Notably, three haplotypes of the short sequences from the Mozambican population were shared with those from populations in the African continent such as Cameroon and DRC. This also suggests the possibility of introduction from those countries (3.8% of import value from DRC) or the same source of introduction; however, analysis with longer sequences is required.

Sequencing of the VSSC gene revealed the presence of the F1534C *kdr* mutation at DIII, with an allele frequency of 46% in the Matola-Rio population. The mutation frequency was 2% in 2020 when first reported in Africa, from Cameroon [32]. Since the F1534C *kdr* mutation was not detected in the study in Cameroon in 2017 [75], this mutation might have developed independently during the 3 years following its introduction as the number of individuals with the mutation needed to be more significant for it to be detected in the earlier study. Similarly, in the present study we analyzed the samples collected 3 years after the first report of *Ae. albopictus* in Mozambique in 2016 [47]. The earlier survey in 2014 could not find this mosquito in Matola-Rio [76]. A control program using insecticides against *Aedes* mosquitoes has never been implemented in Mozambique. As *Ae. albopictus* is a day-biter and exophilic, indoor residual spraying and long-lasting insecticidal nets (LLINs), which are mainly used against anophelines, are less likely affect this mosquito [77, 78] On the other hand, the F1534C mutation has been reported in China and Southeast Asia, where insecticides are intensively used against *Aedes* mosquitoes [29, 30, 34]. This information suggests that the mutation was already present in the mosquito population introduced from abroad.

DNA samples of *Ae. albopictus* from IWIO were not available for the present study, which limits the interpretation of the results from the microsatellite analyses. In addition, our ovitrap collection was operated only once in Matola-Rio, which would underestimate the true haplotype diversity of the present *Ae. albopictus* population in Mozambique.

## Conclusions

Despite the limitations, we have proven that the Matola-Rio *Ae. albopictus* population did not originate from Madagascar or IWIO. Rather, the results from the genetic analyses strongly support the notion that the population was introduced from continental Southeast Asia and a coastal city in China, Hangzhou. The findings of the present study confirmed that the Matola-Rio population had developed the F1534C *kdr* mutation at DIII. The high *kdr* mutation frequency also supports the notion that this population was introduced from abroad.

Since *Ae. albopictus* can adapt to various environments [79], the geographical spread of introduced *Ae. albopictus* may alter the composition of local vector species and the environment of viral disease transmission [80, 81]. In IWIO, *Ae. albopictus* has become predominant in domestic and peridomestic areas where *Aedes aegypti* was once predominant [15, 16, 18, 82]. Although *Ae. albopictus* is thought to play a role in DENV transmission with less competence than *Ae. aegypti* [83], it may play an important role in CHIKV outbreaks and co-infection with DENV in the introduced countries [17, 22, 84, 85]. To prevent further expansion, surveillance and integrated vector control programs should be implemented in the mosquito infested and adjacent areas [86].

### Supplementary Information

**Additional file 1: Table S1.** List of the reference sequences included in the network analyses of COI of *Aedes albopictus*.

**Additional file 2: Table S2.** Indices of genetic diversity for each microsatellite locus of *Aedes albopictus* in Mozambique. A: No. of alleles, Ho: Observed heterozygosity, He: Expected heterozygosity, Gis: Inbreeding coefficient.

**Additional file 3: Table S3.** Microsatellite scores of each locus of* Aedes albopictus *collected in Mozambique.

**Additional file 4: Table S4.** Population pairwise differences (Fst) with the Mozambique population (MOZ). The lower diagonal indicates Fst and the upper diagonal indicates statistical significance.

**Additional file 5: Data S5.** Microsatellite data formats for the analyses used in this study.                

**Additional file 6: R command S6.** R command used for discriminant analysis of principal components (DAPC).

## Data Availability

DNA sequences COI have been submitted to GenBank (LC726376 - LC726425). Microsatellites genotyping data is available in the Additional file.

## Abbreviations

CHIKV: Chikungunya virus
COI: Cytochrome* c* oxidase subunit I
DII: Domain II
DIII: Domain III
DAPC: Discriminant analysis of principal component
DENV: Dengu virus
IWIO: Islands in the Western Indian Ocean
kdr: Knockdown resistance
mtDNA: Mitochondria DNA
RH: Relative humidity
VSSC: Voltage-sensitive sodium channel
ZIKV: Zika virus
