# Supplementary material for: The origin and insecticide resistance of Aedes albopictus mosquitoes established in southern Mozambique
Source: Parasit Vectors. 2024 Jul 8;17:292. doi: 10.1186/s13071-024-06375-6 (PMC11229193; doi:10.1186/s13071-024-06375-6)
Supplement: Supplementary file 1 — Additional file 1: Table S1. List of the reference sequences included in the network analyses of COI of Aedes albopictus. [file 13071_2024_6375_MOESM1_ESM.docx]

**Table S1.** List of the reference sequences included in the network analyses of COI of *Aedes albopictus*.

| Accession numbers | Location | References |
| --- | --- | --- |
| MF148254 | Selangor, Malaysia | Adilah-Amrannudin et al., 2018  (unpublished) |
| MF148260 |  |  |
| MF148261 |  |  |
| MF148263 |  |  |
| MF148267 |  |  |
| MF148270 |  |  |
| MF148279 |  |  |
| MF148282 |  |  |
| MF148286 |  |  |
| KY982342 | Selangor, Malaysia | [57] |
| KY982353 | Selangor, Malaysia | [57] |
| KY982355 | Selangor, Malaysia | [57] |
| KY982357 | Selangor, Malaysia | [57] |
| KY982359 | Selangor, Malaysia | [57] |
| KC690912 | Los Angeles, California, USA / Texas, USA / O’ahu, Hawai’i, USA / Xinzhu, Hsinchu, Taiwan /  Arco, Trentino, Italy (H17) | [50] |
| KC690932 | Italy / New Jersey and Texas, USA (H37) |  |
| KC690940 | Los Angeles, USA (H45) |  |
| KC690951 | Texas, USA (H56) |  |
| MF185675 | Ohio, USA | [53] |
| MH817501 | Novorossiysk, Russia | Fedorova et al., 2018 (unpublished) |
| MH817502 |  |  |
| MH817503 |  |  |
| MH817506 |  |  |
| MH817507 |  |  |
| MH817511 |  |  |
| MH817534 | Chadyschinsk, Russia |  |
| MH817539 | Goryachy Klyuch, Russia |  |
| MH817540 |  |  |
| MH817541 |  |  |
| MH817542 |  |  |
| MH817546 |  |  |
| AB907796 | Costa Rica (H67) | [56] |
| AB907797 | Costa Rica (H68) |  |
| AB907798 | Costa Rica (H69) |  |
| AB907799 | Costa Rica (H70) |  |
| AB907800 | Costa Rica (H71) |  |
| AB907801 | Panama (H72) |  |
| KU738424 | Hangzhou, China | [54] |
| KX383934 | Foshan, China | [60] |
| KC690906 | Xiamen, China (H11) | [50] |
| KC690904 | Xiamen, China (H09) |  |

**Table S1.** (Continued)

| Accession numbers | Location | References |
| --- | --- | --- |
| KC690922 | Singapore (H27) |  |
| MN080729 | Laos / Thailand (H76) | [59] |
| MN080743 | Laos (H90) |  |
| KX383926 | Thailand | [60] |
| LC591863 | Ronong, Thailand | [58] |
| MW279069 | Mallorca, Spain | [55] |
| KX383924 | Brazil | [60] |
| KX383927 | Greece |  |
| HQ622905 | Reunion Island | [11] |
| HQ622907 |  |  |
| HQ622908 |  |  |
| HQ622909 |  |  |
| HQ622910 |  |  |
| HQ622911 |  |  |
| HQ622912 |  |  |
| HQ622913 |  |  |
| HQ622914 |  |  |
| HQ622915 |  |  |
| HQ622916 |  |  |
| HQ622917 |  |  |
| HQ622918 |  |  |
| HQ622919 |  |  |
| HQ622920 |  |  |
| HQ622921 |  |  |
| HQ622922 |  |  |
| HQ622923 |  |  |
| HQ622924 |  |  |
| HQ622925 |  |  |
| HQ622926 |  |  |
| HQ622927 |  |  |
| HQ622928 |  |  |
| HQ623004 | Glorioso Islands |  |
| HQ623005 |  |  |
| HQ623006 |  |  |
| HQ622906 | Madagascar |  |
| HQ622959 |  |  |
| HQ622960 |  |  |
| HQ622961 |  |  |
| HQ622962 |  |  |
| HQ622964 |  |  |
| HQ622965 |  |  |
| HQ622966 |  |  |
| HQ622967 |  |  |
| HQ622968 |  |  |
| HQ622969 |  |  |
| HQ622970 |  |  |
| HQ622971 |  |  |

**Table S1.** (Continued)

| Accession numbers | Location | References |
| --- | --- | --- |
| HQ622972 | Madagascar | [11] |
| HQ622973 |  |  |
| HQ622974 |  |  |
| HQ622975 |  |  |
| HQ622976 |  |  |
| HQ622977 |  |  |
| HQ622978 |  |  |
| HQ622979 |  |  |
| HQ622929 | Mauritius Island |  |
| HQ622930 |  |  |
| HQ622931 |  |  |
| HQ622932 |  |  |
| HQ622986 |  |  |
| HQ622987 |  |  |
| HQ622988 |  |  |
| HQ622980 |  |  |
| HQ622981 |  |  |
| HQ622982 |  |  |
| HQ622983 |  |  |
| HQ622984 |  |  |
| HQ622985 |  |  |
| HQ622933 |  |  |
| HQ622934 |  |  |
| HQ622935 |  |  |
| HQ622936 |  |  |
| HQ622937 |  |  |
| HQ622938 |  |  |
| HQ622939 |  |  |
| HQ622940 |  |  |
| HQ622941 |  |  |
| HQ622942 |  |  |
| HQ622943 |  |  |
| HQ622944 |  |  |
| HQ622945 |  |  |
| HQ622946 |  |  |
| HQ622947 |  |  |
| HQ622948 |  |  |
| HQ622949 |  |  |
| HQ622950 |  |  |
| HQ622951 |  |  |
| HQ622952 |  |  |
| HQ622953 |  |  |
| HQ622954 |  |  |
| HQ622955 |  |  |
| HQ622956 |  |  |
| HQ622957 |  |  |
| HQ622958 |  |  |
| HQ622989 | Rodrigues |  |

**Table S1.** (Continued)

| Accession numbers | Location | References |
| --- | --- | --- |
| HQ622990 | Rodrigues | [11] |
| HQ622991 |  |  |
| HQ622992 |  |  |
| HQ622993 |  |  |
| HQ622994 |  |  |
| HQ622995 |  |  |
| HQ622996 | Seychelles Islands |  |
| HQ622997 |  |  |
| HQ622998 |  |  |
| HQ622999 |  |  |
| HQ623000 |  |  |
| HQ623001 |  |  |
| HQ623002 |  |  |
| HQ623003 |  |  |
| HQ623004 |  |  |
| HQ398900 | Vietnam | [62] |
| HQ398901 |  |  |
| JX912500 | Lebanon | [63] |
| KX383930 | Albania | [61] |
| KX383931 |  |  |
| KX383932 | Greece |  |
| MH025948 | Republic of the Congo | [45] |
| MH025949 |  |  |
| MH025950 |  |  |
| KU522419 | Morocco | [64] |
| KU522420 |  |  |
| KU522421 |  |  |
| KX495911 | Vietnam | [65] |
| MH921568 | Cameroon | [66] |
| MH921569 |  |  |
| MH921570 |  |  |
| MH921571 |  |  |
| MH921572 |  |  |
| MT345350 | Democratic Republic of the Congo | [67] |
| MT345351 |  |  |
| MT345352 |  |  |
| MT345353 |  |  |
| MT345354 |  |  |
| MT345355 |  |  |
| MT345356 |  |  |
| MT345358 |  |  |
| MT345359 |  |  |
| MT345364 |  |  |
| MT345365 |  |  |
| MT345366 |  |  |
| MT345367 |  |  |
| MT345368 |  |  |
| MT345369 |  |  |

**Table S1.** (Continued)

| Accession numbers | Location | References |
| --- | --- | --- |
| MT345370 | Democratic Republic of the Congo | [67] |
| MT345371 |  |  |
| MT345372 |  |  |
| MT345373 |  |  |
| MT345374 |  |  |
| MT345375 |  |  |
| MT345376 |  |  |
| MT345377 |  |  |
| MT345378 |  |  |
| MT345379 |  |  |
| MT345380 |  |  |
| MT345381 |  |  |
| MT345382 |  |  |
| MT345383 |  |  |
| MT345384 |  |  |
| MT345385 |  |  |
| MT345386 |  |  |
| MT345387 |  |  |
| MT345388 |  |  |
| MT345390 |  |  |
