# Supplementary material for: The origin and insecticide resistance of Aedes albopictus mosquitoes established in southern Mozambique
Source: Parasit Vectors. 2024 Jul 8;17:292. doi: 10.1186/s13071-024-06375-6 (PMC11229193; doi:10.1186/s13071-024-06375-6)
Supplement: Supplementary file 2 — Additional file 2: Table S2. Indices of genetic diversity for each microsatellite locus of Aedes albopictus in Mozambique. A: No. of alleles, Ho: Observed heterozygosity, He: Expected heterozygosity, Gis: Inbreeding coefficient. [file 13071_2024_6375_MOESM2_ESM.docx]

**Table S2.** Indices of genetic diversity for each microsatellite locus of *Aedes albopictus* in Mozambique. A: No. of alleles, Ho: Observed heterozygosity, He: Expected heterozygosity, Gis: Inbreeding coefficient.

| Locus | A | Ho | He | Gis |
| --- | --- | --- | --- | --- |
| DI4 | 3 | 0.189 | 0.311 | 0.391 |
| DI6 | 6 | 0.62 | 0.742 | 0.165 |
| DI3 | 4 | 0.52 | 0.564 | 0.077 |
| TRI6 | 6 | 0.553 | 0.78 | 0.29 |
| TRI18 | 8 | 0.62 | 0.708 | 0.124 |
| TRI20 | 6 | 0.72 | 0.686 | -0.049 |
| TRI21 | 5 | 0.32 | 0.669 | 0.522 |
| TRI25 | 5 | 0.62 | 0.57 | -0.088 |
| TRI33 | 7 | 0.78 | 0.765 | -0.019 |
| TRI44 | 6 | 0.265 | 0.783 | 0.661 |
| TRI44 | 6 | 0.3 | 0.398 | 0.245 |
| TRI45 | 4 | 0.72 | 0.739 | 0.025 |
| TRI46 | 7 | 0.28 | 0.789 | 0.645 |
| Total | 5.615 | 0.501 | 0.654 | 0.235 |
